# Nigeria’s post-COVID-19 vaccine manufacturing ambitions: Opportunities and regional implications

**DOI:** 10.4102/jphia.v17i1.1560

**Published:** 2026-02-02

**Authors:** Oyeronke Oyebanji, Olufunke Falade, Frederik Kristensen, David Heymann, Beate Kampmann

**Affiliations:** 1 London School of Hygiene and Tropical Medicine, London, United Kingdom; 2 Nigeria Presidential Unlocking Healthcare Value Chain, Abuja, Nigeria; 3 Regional Vaccine Manufacturing Collaborative, Oslo, Norway

**Keywords:** COVID-19, pandemic preparedness, regional cooperation, vaccine equity, vaccine manufacturing

## Abstract

**Background:**

Global health emergencies consistently expose and exacerbate vaccine inequities, with high-income countries prioritising their populations and leaving low- and middle-income countries (LMICs) facing delays and shortages. Diseases primarily affecting LMICs receive limited attention from global vaccine developers because of perceived low market value and limited financial return, further entrenching these disparities. Africa’s limited investment in vaccine manufacturing has heightened its vulnerability during outbreaks, including yellow fever, Ebola, meningococcal meningitis, mpox, and most recently, coronavirus disease 2019 (COVID-19).

**Aim:**

This study examines the barriers to vaccine equity in Africa and critically analyses Nigeria’s post-COVID-19 efforts to establish a viable, local vaccine manufacturing ecosystem.

**Setting:**

The research focuses on Nigeria within the broader African vaccine manufacturing landscape, using it as a case study to explore both national and continental dynamics.

**Methods:**

The study draws on document analysis of strategic plans, regulatory reports, and partnership announcements, complemented by qualitative insights from key informant interviews with stakeholders involved in vaccine policy, regulation, and production in Nigeria and across West Africa.

**Results:**

Post-pandemic momentum has catalysed significant shifts: Nigeria has developed a national vaccine manufacturing strategy and secured international partnerships and financing commitments. The regulatory authority, National Agency for Food and Drug Administration and Control (NAFDAC), achieved the World Health Organization (WHO) Maturity Level 3 status, marking critical progress. Nonetheless, persistent gaps remain in research and development capacity, workforce development, regulatory agility, and infrastructure readiness.

**Conclusion:**

While Nigeria has made notable progress since the COVID-19 pandemic, sustainable vaccine manufacturing requires long-term investment in research and development, policy reform, skills development, and regional cooperation. Failure to address these challenges systematically risks undermining current gains.

**Contribution:**

This article provides insights to support ongoing and future investments in Nigeria’s vaccine manufacturing sector, guiding government policy, international partnerships, and potential investors.

## Introduction

During the coronavirus disease 2019 (COVID-19) pandemic, countries faced a stark inequity in access to health products, including vaccines, therapeutics and diagnostics.^1,2,3^ As the pandemic spread, there were widespread disruptions in supply chains, leading to shortages of essential vaccines needed to control the spread, medical supplies and equipment in various parts of the world.^4,5^ The concentration of manufacturing facilities in specific regions exacerbated these challenges, as lockdowns, travel restrictions and logistical bottlenecks impeded the smooth flow of goods, including vaccines.^6^ This heightened the realisation that reliance on a few key manufacturing hubs leaves the global community vulnerable to unforeseen disruptions. Since then, there have been recommendations and initiatives to decentralise manufacturing capabilities and establish a more geographically diverse network of production facilities for vaccines and other health products.^7,8^ This article situates the COVID-19 vaccine inequity experience within a broader historical context of health crises, exposing vulnerabilities in vaccine manufacturing.

Vaccine inequity, defined as unequal distribution and access to vaccines in certain regions, remains an important issue, especially during global health emergencies, most recently exemplified by COVID-19 and mpox.^9^ Key factors driving inequity include vaccine nationalism, intellectual property restrictions and limited access to technical know-how, insufficient manufacturing capacity and fragile healthcare systems.^10^

There are several reasons why vaccine inequity is most pronounced during emergencies. Firstly, high-income countries (HICs) typically secure early access to vaccines, leaving low- and middle-income countries (LMICs) facing delays and shortages. For example, despite the urgent need for 10 million mpox vaccine doses in Africa, as assessed by the Africa Centres for Disease Control and Prevention (CDC) in 2024,^9^ less than 1 million doses were allocated to African countries, with donations from the United States (US), Canada, the European Union and Japan.^11^ It is also unclear how many African countries have procured doses themselves, especially given the high cost.^9^ The Democratic Republic of Congo (DRC), one of the worst-affected countries, only commenced a vaccination programme in October 2024, long after the virus had spread across the region. This mirrors the challenges faced during COVID-19 when resource-limited countries struggled to secure vaccines on time.

Additionally, because of limited market opportunities, vaccine developers and manufacturers invest limited resources, if any, in vaccines to combat diseases primarily affecting LMICs, such as mpox and Lassa fever, exacerbating access inequity to potentially life-saving interventions.^12^ High-income countries’ developers and manufacturers typically prioritise diseases impacting their markets with higher profit margins, leaving LMICs without vaccines for regional diseases or reliant on donations and delayed access.^13^

In 2021, the African Union (AU) set an ambitious goal – ‘to develop, produce, and supply more than 60% of the vaccine doses required on the continent by 2040’, with 10% of these doses produced on the continent by 2025.^14^ Some progress is evident with the construction of messenger ribonucleic acid (mRNA) vaccine manufacturing facilities in Rwanda and active technology transfer initiatives in Morocco, Egypt, Senegal and South Africa.^15^ Countries such as Kenya, Ethiopia, Ghana, Nigeria and others have recently developed strategies and are working towards action. According to a 2024 survey by Africa CDC and PATH, an estimate shows 25 active vaccine projects across Africa: five manufacturers have commercial-scale facilities with ongoing or completed technology transfers, five have such facilities but have not yet secured technology transfers and 15 are in earlier development stages.^16^

This article explores progress in vaccine manufacturing across Africa post COVID-19, focusing on Nigeria’s strategy. It provides an original analysis of Nigeria’s vaccine manufacturing ambitions within the context of health security and post-COVID-19 resilience, drawing on policy documents, stakeholder interviews and regional initiatives. It assesses implications for epidemic preparedness and regional manufacturing coordination.

## Research methods and design

This study employed a qualitative design to explore Nigeria’s progress and strategy for local vaccine manufacturing in the post-COVID-19 context, situating it within Africa’s broader health security and regional manufacturing agenda.

Data collection combined two main approaches: semi-structured interviews with key national and regional stakeholders and document analysis of relevant national strategies and regional initiatives. Reviewed documents included Nigeria’s National Vaccine Policy, the National Plan for Vaccine Development and Manufacturing, COVID-19 recovery and health security frameworks and continental strategies such as the African Union’s PHAHM (Platform for Harmonised African Health Products Manufacturing) Framework for Action.

Semi-structured interviews were conducted with policymakers, regulatory officials, local pharmaceutical manufacturers and representatives from regional and international bodies supporting vaccine manufacturing and epidemic preparedness. Participants were purposively selected for their direct roles in policy development, implementation or coordination.

Data were analysed thematically using two conceptual frameworks: the Four-by-Four Framework, which examines how informal institutions (social norms), formal institutions (policies and regulations), organisational structures (governance and missions), and everyday exchange (service delivery) enable or constrain innovation; and the Control Knobs Framework, which considers how financing, payment systems, organisational arrangements, regulation, and stakeholder behaviours interact to shape health system outcomes.

Findings were triangulated across policy documents and stakeholder insights to assess Nigeria’s institutional readiness, alignment with continental vaccine manufacturing goals, and implications for future epidemic preparedness and regional manufacturing coordination.

### Ethical considerations

Ethical clearance to conduct this study was obtained from the National Health Research Ethics Committee of Nigeria (NHREC) (No. NHREC/01/01/2007-27/09/2024) and the London School of Hygiene and Tropical Medicine Research Ethics Committee (No. 31269). Participation in the study was entirely voluntary. Before taking part, participants were provided with a detailed information sheet outlining the purpose of the study, the methods involved, potential risks and benefits, and their rights as participants. Participants were given the opportunity to ask questions and discuss the study before signing a consent form.

## Results

### Consequences of Africa’s limited investment in vaccine manufacturing and R&D

Despite being home to more than 1.2 billion people, Africa accounts for less than 1% of global vaccine manufacturing.^14^ The continent’s reliance on imported vaccines and limited investment in manufacturing and research and development (R&D) has left it highly vulnerable to disease outbreaks. The following examples illustrate major outbreaks whose impact could have been mitigated with improved vaccine access and development capabilities.

Yellow fever has been endemic to several African countries for centuries. Although the virus was first identified by the Institut Pasteur in Dakar (IPD), Senegal, which also produces vaccines, production capacity remains insufficient to meet the demands of endemic countries.^17^ While a highly effective vaccine has been available since the 1970s, poor vaccination coverage has led to recurring outbreaks. A large outbreak in Angola from 2015 to 2016^18^ exposed Africa’s limited vaccine access.^18,19^ At that time, yellow fever vaccine production was limited to a few global manufacturers, most located outside Africa.^19^ As a result, African countries often relied on delayed and insufficient supplies of yellow fever vaccines from international emergency stockpiles managed by the World Health Organization (WHO) and United Nations Children’s Fund (UNICEF).^20,21^ The response was additionally hindered by weak infrastructure and inadequate health systems that struggled to deliver yellow fever vaccines quickly.^18^

The 2014–2016 Ebola epidemic equally highlighted vaccine inequity: while vaccine development was expedited in response to the outbreak,^22^ there were significant delays in conducting vaccine trials, and the experimental vaccine was limited to emergency use under highly controlled conditions.^23^ This meant that the countries most affected – Sierra Leone, Guinea and Liberia – could not access vaccines in time to halt the spread of the disease. The lack of industry incentives to invest in this disease, with sporadic, relatively small outbreaks and no clear buyer, highlighted how the market naturally prioritises profitability over public health needs, sometimes labelled a ‘market failure’. This reflects the inherent limitations of traditional market dynamics. International partnerships for vaccine R&D, including institutions such as the Coalition for Epidemic Innovations (CEPI), were established in recognition of this gap and response to the crisis.^24^ However, the delay in developing an Ebola vaccine underscored how Africa’s dependence on external actors left the continent vulnerable to outbreaks.

The 2009 H1N1 influenza pandemic (commonly known as swine flu) again demonstrated how global health emergencies disproportionately affect LMICs. Despite the global threat posed by the virus, vaccine access was highly uneven.^25^ Wealthy countries quickly secured the majority of available doses through advanced purchase agreements with pharmaceutical companies, leaving African countries and other LMICs with limited or no access to vaccines during the pandemic’s critical early stages.^26^

### Root causes of vaccine inequity

A critical issue for Africa has been the near-total reliance on a manufacturing industry for vaccines outside of the continent. This lack of investment in domestic manufacturing capacity has led to a situation where African countries are heavily dependent on international procurement systems such as Gavi the Vaccine Alliance^27^ or on donations during public health emergencies, as was seen during COVID-19 and now with mpox.^28^ When global demand surged during crises, such as with the H1N1 influenza or the COVID-19 pandemic, African countries were often last to receive vaccines.

Even when vaccines were available, financial barriers made it difficult for many African countries to secure sufficient quantities to meet their needs.^27,29^ Vaccines are often expensive, and LMICs, particularly those in Africa, faced budgetary constraints that limited their ability to purchase vaccines independently.^30^ In addition, many African countries were and continue to be dependent on donor funding and international aid to meet their health needs.^31,32^ This reliance on external funding sources has created vulnerabilities, as donor priorities may not always align with the health needs of African countries.^33^

The ability to effectively distribute and administer vaccines is dependent on a well-functioning health system, including cold chain infrastructure, trained healthcare workers, and access to remote areas.^34^ Many African countries have historically struggled with weak healthcare infrastructure, affecting their ability to execute vaccination campaigns swiftly and efficiently. This issue was particularly pronounced during the yellow fever and Ebola outbreaks between 2014 and 2016, where inadequate infrastructure delayed the timely distribution of vaccines to affected populations.^21,35,36^

**FIGURE 1:**
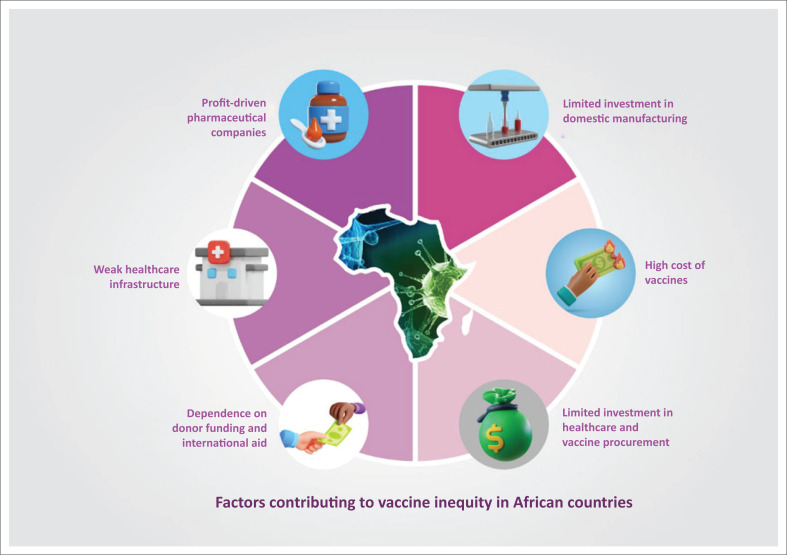
Factors contributing to vaccine inequity in African countries.

Global health governance structures, including those governing vaccine procurement and distribution, face challenges despite their intents. Advanced economies often enter into agreements with pharmaceutical companies to secure large quantities of vaccines, leaving LMICs to rely on mechanisms such as COVAX (for COVID-19) or international donations for access.^37^ The African Union’s Vaccine Acquisition Task Team (AVATT) faced supply constraints, leaving the total doses delivered unclear. This highlights the need for robust demand-pooling systems during ‘peacetime’. The pharmaceutical industry, often driven by profit incentives, tends to prioritise diseases affecting HICs, leading to a ‘market failure’ in addressing diseases that disproportionately affect African populations, such as malaria, HIV and Ebola.^38^ These factors are visualised in Figure 1.^38^

### Post-COVID-19 initiatives: Advocating for local vaccine manufacturing in Africa

Establishing local vaccine manufacturing potentially offers numerous advantages, such as enhanced self-sufficiency, quicker responses to outbreaks, and reduced reliance on imports.^12^ However, significant challenges remain, including infrastructure limitations, regulatory hurdles, a lack of technical expertise, interest in sharing intellectual property and tech transfer, and the need for sustainable financing.^12^ Manufacturing alone cannot resolve issues related to distribution, equitable access, and integration into health systems, or market sustainability.

In 2021, the WHO established an mRNA vaccine technology transfer programme to help LMICs establish mRNA manufacturing capacity, starting with COVID-19 vaccines.^39^ In 2021, the Africa Centres for Disease Control (Africa CDC) established *the Partnership for Africa Vaccine Manufacturing* (PAVM) (renamed and expanded to PHAHM – Platform for Harmonized African Health Products Manufacturing in April 2024) as a continental strategy to scale up vaccine manufacturing capabilities.^14^

In June 2024, Gavi announced the launch of the African Vaccine Manufacturing Accelerator (AVMA) as a financing mechanism designed to allocate up to $1 billion over 10 years towards expediting the growth of commercially sustainable vaccine manufacturing in Africa.^40^ Beyond this, countries such as Nigeria, Rwanda, Senegal and South Africa have announced partnerships to advance local vaccine manufacturing.^41,42^

South Africa has emerged as a front-runner in vaccine production, mainly through the efforts of Aspen Pharmacare, BioVac and with Afrigen as the host of the WHO’s mRNA Technology Transfer Hub.^39^ In September 2024, Aspen announced plans to manufacture mpox vaccines at its facility.^43^ Despite these efforts, there have been challenges, including the refusal of high-income country-based companies to share intellectual property with local vaccine manufacturers. The government’s decision in 2023 to purchase pneumococcal vaccines from India, despite having a local manufacturer capable of production, highlights the significant role of cost-competitiveness in vaccine procurement.^44^ This underscores how economic factors, such as competitive pricing, can undermine the viability of local manufacturing, even when capacity exists.

Senegal and Egypt have long-established institutions advancing vaccine production, although they face challenges in scaling up their capabilities. Institut Pasteur in Dakar has been a critical player in vaccine production for yellow fever vaccines for decades, with plans to commemorate its 100th anniversary in 2024. It is the only African manufacturer prequalified by the WHO to produce yellow fever vaccines.^19^ With funding from CEPI, the European Union and other donors, IPD is building new facilities to expand its vaccine production capacity. Senegal still faces challenges, including ensuring sustainable funding, developing local expertise, and overcoming the national regulatory authority’s limited capacity.^14^

In Egypt, the government has focused on expanding its domestic vaccine production capacity through its state-owned company, Vacsera, established in 1881 and with fill and finish capabilities.^45^ Egypt partnered with Sinovac to produce COVID-19 vaccines locally, which allowed the country to reduce its reliance on imports and supply vaccines domestically and regionally.^45^ Egypt also aims to produce pentavalent and hepatitis vaccines through a partnership with Serum Institute of India.^46^ While Egypt’s vaccine manufacturing initiatives have been successful, challenges include scaling up production to meet regional demand and ensuring that locally produced vaccines meet international quality standards.^30^

### Nigeria’s post-COVID-19 vaccine manufacturing plans

In Nigeria, a country with a large and diverse population, infectious diseases and emerging epidemics pose significant health challenges.^47^ The Africa CDC’s PHAHM framework for action recognises that the number of countries that would become ‘self-procuring’ for vaccines is expected to grow in the next decade as more countries, including highly populated countries such as Nigeria, are expected to transition from Gavi support.^14^ Despite Africa’s large population size and high burden of disease, there is minimal capacity for vaccine research and development and manufacturing in Nigeria.^46^

Nigeria’s limited vaccine research and development capacity could be linked to several challenges. These include the limited domestic funding available for research, inadequate infrastructure, limited expertise, including for regulatory approvals, the limited number of biotechs, and concerns regarding data quality and integrity, among others.^48,49,50^ In 2021, a National Vaccine Policy was established to address some of these issues, including providing funding for R&D, and in 2023, a National Plan for Vaccine R&D and Local Production was published.^51^

Among its goals and timelines, this plan highlights the intention to establish (1) a lab-scale plant focused on mRNA technology to contribute to clinical advancements tailored to local diseases, with an estimated completion timeline of 3 years to 4 years; (2) fill and finish capacity for non-live vaccine products within 2 years to 3 years; (3) a full-scale domestic manufacturing plant, including drug substance production and fill and finish capabilities for live attenuated vaccines, which is anticipated to be operational within 3 years to 4 years.

In September 2023, Nigeria announced the ‘Presidential Unlocking Healthcare Value-Chain Initiative’, or PVAC, representing the government’s plan for medical industrialisation in Nigeria to enable collaboration between the public and private sectors to drive innovation and enhance healthcare product development. The goal, according to the government, is to transform the Nigerian health sector by boosting local production of health products and technologies, improving job quality and quantity in the healthcare value chain, and reducing medical tourism. Since the announcement of this initiative, the Government of Nigeria has announced investments worth $240 million from an unnamed Brazilian pharmaceutical company for local manufacturing of pharmaceuticals, with Belgian biotech UniverCells, and a $1bn ‘funding line’ for viable health investments from the African Export-Import Bank.^52^

Following the COVID-19 pandemic, the European Union launched the ‘Team Europe Initiative on Manufacturing and Access to Vaccines, Medicines, and Health Technologies (MAV+)’. This initiative aims to strengthen African countries’ local pharmaceutical systems and manufacturing capacities. Under MAV+, the Government of Nigeria and the EU signed a cooperation agreement through which the EU will provide €18m within an unannounced timeline to support Nigeria’s efforts to enhance the local production of vaccines and other health products.^53^

According to a 2022 report by the Wellcome Trust, Nigeria has two companies with varying R&D and manufacturing capacities.^54^ Both companies, Innovative Biotech and BioVaccines, were founded in 2005. Innovative Biotech has been reported to possess R&D capabilities for Ebola, yellow fever, COVID-19, and HPV,^54^ as well as fill and finish capacity. However, beyond results from surveys and reports, there remains a lack of evidence of production, including scientific research outputs, to substantiate these claims. BioVaccines Nigeria Limited is a public–private venture jointly owned by the Federal Government of Nigeria and May and Baker Nigeria PLC. However, operations have not begun because of limited access to funds. BioVaccines is described as an ‘aspirational partner’ of the WHO mRNA technology transfer Hub compared to other established spokes. It is establishing a facility to advance its vaccine manufacturing goals.

Regulatory capacity is an important aspect of the vaccine manufacturing ecosystem, as insufficient regulatory oversight can lead to compromised product (vaccine) quality, adverse effects, and jeopardise public trust.^55^ In addition, vaccine procurement agencies such as Gavi require vaccines to receive prequalification from the WHO, which requires the national regulatory agencies of countries where vaccine manufacturing occurs to operate at a minimum of Maturity Level 3 for vaccine production.^55^ Therefore, understanding the country’s regulatory capacity is essential. The National Agency for Food and Drug Administration and Control (NAFDAC) is Nigeria’s national medicines regulatory agency, responsible for ensuring the safety, quality, and efficacy of vaccines and other pharmaceutical products in Nigeria. As the primary regulatory body, NAFDAC’s policies and activities significantly influence the landscape of vaccine manufacturing and distribution within the country. According to a WHO-led survey in 2022, pharmaceutical sector stakeholders have varying levels of confidence in NAFDAC’s regulatory pathways for vaccine registration and lot release, with 42.8% expressing high confidence. Similarly, 40% of respondents acknowledged the existence of defined policies and procedures for vaccine regulation, although 37.1% were uncertain.^56^ These findings suggest a need for greater visibility and awareness of NAFDAC’s regulatory systems to support vaccine manufacturing initiatives. In addition, the review suggests that there is limited understanding and implementation of regulatory reliance and convergence mechanisms within NAFDAC.

In 2022, the WHO certified NAFDAC as operating at Maturity Level 3 for medicines and vaccines without production. This means Nigeria has a functional regulatory system for medicines and imported vaccines. However, a further evaluation will be required for local vaccine manufacturing. Further research would need to investigate the barriers and facilitators to implementing the requirements to reach regulatory competencies, evaluate how other countries have successfully implemented regulatory reliance and convergence mechanisms, and identify what lessons can be applied to the Nigerian context.

Firstly, Nigeria’s renewed focus on local vaccine manufacturing could be described as timely and relevant as Nigeria prepares to transition from Gavi’s support by 2028. It highlights the urgency of developing sustainable, self-reliant vaccine manufacturing and procurement mechanisms. Secondly, through PVAC, Nigeria is considering strategies and focus areas for investment to advance its local vaccine manufacturing goals. Thirdly, given Nigeria’s population size and vaccine market, the success (or lack of) of its local vaccine manufacturing strategy could impact initiatives such as the Africa CDC’s PHAHM.

While this article focuses on human vaccine production, it is important to recognise Nigeria’s existing capacity in animal vaccine production. The National Veterinary Research Institute (NVRI) has been a key player in veterinary vaccine production for over seven decades, developing 22 bacterial and viral vaccines using conventional technologies like embryonated chicken eggs, cell culture and bacterial culture.^57^ The NVRI’s operations are evenly split between research and vaccine production, and the institute is expanding into modern biotechnologies, including deoxyribonucleic acid (DNA) and mRNA technologies.^56^

## Discussion – What next for Nigeria?

Vaccine availability and accessibility are complex challenges influenced by political, economic and socio-cultural factors. Political considerations such as government policies, regulations and international agreements significantly influence vaccine manufacturing and distribution. Nigeria’s dependence on Gavi for routine vaccination needs and its upcoming graduation, which requires readiness to self-procure vaccines, are contributing to the urgency of local manufacturing capacity.

For vaccine manufacturing plans to succeed, robust policies, investment in R&D, a skilled workforce, regulatory strengthening, market analysis and partnerships with global stakeholders must support the efforts. Despite ambitious plans through initiatives such a PVAC and the National Vaccine Policy, policy implementation and investment gaps remain. The closure of the Federal Vaccine Production Laboratory in Lagos in 1991 underscores the importance of learning from past failures to ensure success in future efforts.

Regulatory capacity remains a critical challenge. While NAFDAC’s attainment of Maturity Level 3 is a positive step, strengthening its organisational and regulatory competencies to support local vaccine manufacturing is urgent. The limited understanding and implementation of regulatory reliance and convergence mechanisms indicate a need for improved international collaboration, adaptation of models from other countries and capacity building.

Nigeria’s current R&D capacity, as demonstrated by institutions such as the National Institute for Pharmaceutical Research and Development (NIPRD) and the Nigerian Institute of Medical Research (NIMR), shows potential but suffers from limited published research and evidence to support local manufacturing ambitions. Understanding the gaps and strengthening R&D capabilities, particularly in emerging technologies such as mRNA, will be crucial for advancing Nigeria’s vaccine manufacturing ambitions.

Local vaccine manufacturing can offer economic and societal benefits, including job creation, economic growth and reduced import dependency, contributing to health security and resilience. However, government commitment is critical to enabling regulation, infrastructure, market demand and equitable technology transfer. Without this, progress may be hindered, leading to wasted resources. Aspen Pharmacare’s experience in South Africa, which is faced with a lack of orders, including from the South African government, underscores the need for such a comprehensive approach.

Any strategy to enhance local vaccine manufacturing must be tailored to Nigeria’s unique socio-political and economic contexts, including infrastructure gaps, workforce shortages and in-country disparities. Engaging local communities and stakeholders in the planning and implementation process will be crucial for ensuring that the benefits of these initiatives are equitably distributed and that they address the most pressing health needs of the population.

## Conclusion

Nigeria’s progress in vaccine manufacturing could have significant implications beyond its borders. As Africa’s most populous country and with a significantly high burden of vaccine-preventable diseases and risk of infectious disease outbreaks,^47^ Nigeria’s experience in this area could serve as a model for other African countries, potentially influencing regional strategies and policies. The PHAHM and other continental initiatives stand to benefit from Nigeria’s experiences and outcomes, underscoring the interconnected nature of these efforts.

The progress and sustainability of Nigeria’s vaccine manufacturing initiatives will depend on several factors: effective policy development and implementation; an assured market, whether local or international; sustained government commitment, leveraging national, regional and global support; and the ability to attract and retain investment. It will also involve a long-term strategy of creating a supportive environment for public–private partnerships, essential for long-term success. The Regional Vaccine Manufacturing Collaborative set out a framework for the various elements needed to establish sustainable manufacturing that can be applied to Nigeria.^58^

Finally, a systematic assessment of the institutional enablers and barriers to Nigeria’s vaccine manufacturing plans, with detailed strategies to address them, should be considered. This feasibility study should ensure alignment with practical realities and global best practices by evaluating factors such as technical expertise, infrastructure readiness, regulatory frameworks, and financial sustainability or market demand to determine the viability of local vaccine manufacturing.

With the right strategies and sustained commitment, Nigeria has the potential to become a key player in global vaccine manufacturing, contributing to a more equitable and resilient health landscape.

## References

[1] Gleeson D, Townsend B, Tenni BF, Phillips T. Global inequities in access to COVID-19 health products and technologies: A political economy analysis. Health Place. 2023;83:103051. 10.1016/j.healthplace.2023.103051 37379732 PMC10247888

[2] Mammen M, Narasimhan V, Kuntz R, Lewis-Hall F, Poul M, Schechter A. Health product manufacturers and innovators COVID-19 impact assessment: Lessons learned and compelling needs. NAM Perspect. 2022;2022:10.31478/202201b. 10.31478/202201b PMC8970224 35402857

[3] Luyckx VA. Health inequality and inequity during the COVID-19 pandemic. Nephrol Dial Transplant. 2023;38(11):2417–2419. 10.1093/ndt/gfad119 37309023

[4] Panwar R, Pinkse J, De Marchi V. The future of global supply chains in a post-COVID-19 world. Calif Mgmt Rev. 2022;64(2):5–23. 10.1177/00081256211073355

[5] De Marchi V, Alford M. State policies and upgrading in global value chains: A systematic literature review. J Int Bus Policy. 2022;5(1):88–111. 10.1057/s42214-021-00107-8

[6] Koller CN, Schwerzmann CJ, Suzanne A, et al. Addressing different needs: The challenges faced by india as the largest vaccine manufacturer while conducting the world’s biggest COVID-19 vaccination campaign. Epidemiol. 2021;2(3):454–470. 10.3390/epidemiologia2030032 PMC9620944 36417236

[7] Dzau VJ, Balatbat CA, Offodile AC. Closing the global vaccine equity gap: Equitably distributed manufacturing. Lancet. 2022;399(10339):1924–1926. 10.1016/S0140-6736(22)00793-0 35533706 PMC9075857

[8] Sell TK, Gastfriend D, Watson M, et al. Building the global vaccine manufacturing capacity needed to respond to pandemics. Vaccine. 2021;39(12): 1667. 10.1016/j.vaccine.2021.02.017 33640143 PMC7903906

[9] Gostin LO, Jha AK, Finch A. The Mpox global health emergency – A time for solidarity and equity. N Engl J Med [serial online]. 2024 [cited 2024 Oct 03]. Available from: https://www.nejm.org/doi/full/10.1056/NEJMp2410395 10.1056/NEJMp2410395 39197094

[10] Ferranna M. Causes and costs of global COVID-19 vaccine inequity. Semin Immunopathol. 2023; 45(4–6):469–480. 10.1007/s00281-023-00998-0 37870569 PMC11136847

[11] Vaccine doses allocated to 9 African countries hardest hit by mpox surge [homepage on the Internet]. [cited 2024 Nov 08]. Available from: https://www.who.int/news/item/06-11-2024-vaccine-doses-allocated-to-9-african-countries-hardest-hit-by-mpox-surge

[12] Hayman B, Suri RK. Sustainable vaccine manufacturing in low- and middle-Income countries. Vaccine. 2022;40(50):7288–7304. 10.1016/j.vaccine.2022.10.044 36334966

[13] Smith DRM, Turner J, Fahr P, et al. Health and economic impacts of Lassa vaccination campaigns in West Africa. Nat Med 2024 [serial online]. 2024 [cited 2024 Oct 13];1–10. Available from: https://www.nature.com/articles/s41591-024-03232-y 10.1038/s41591-024-03232-y PMC11645265 39198710

[14] Africa Centres for Disease Control and Prevention (Africa CDC). Partnerships for African Vaccine Manufacturing (PAVM): Framework for action [homepage on the Internet]. Addis Ababa: Africa CDC; 2022 [cited 2025 Jan 25]. Available from: https://africacdc.org/download/partnerships-for-african-vaccine-manufacturing-pavm-framework-for-action/

[15] Africa Centres for Disease Control and Prevention (Africa CDC). The change in African vaccine manufacturing landscape [homepage on the Internet]. 2024 [cited 2025 Feb 16]. Available from: https://africacdc.org/wp-content/uploads/2024/11/The-change-in-African-vaccine-manufacturing-landscape-.pdf

[16] PATH. How has the African vaccine manufacturing landscape changed in the last year? [homepage on the Internet]. PATH; 2024 [cited 2024 Nov 13]. Available from: https://www.path.org/our-impact/articles/how-has-the-african-vaccine-manufacturing-landscape-changed-in-the-last-year/

[17] World Health Organization. A global strategy to eliminate yellow fever epidemics 2017–2026 [homepage on the Internet]. 2018 [cited 2025 Feb 16]. Available from: www.who.int/csr/disease/yellowfev/eye-strategy/en/

[18] Kraemer MUG, Faria NR, Reiner RC, et al. Spread of yellow fever virus outbreak in Angola and the Democratic Republic of the Congo 2015–16: A modelling study. Lancet Infect Dis. 2017;17(3):330–338. 10.1016/S1473-3099(16)30513-8 28017559 PMC5332542

[19] Barrett ADT. Yellow fever in angola and beyond – The problem of vaccine supply and demand. N Engl J Med. 2016;375(4):301–303. 10.1056/NEJMp1606997 27276108

[20] Monath TP, Woodall JP, Gubler DJ, et al. Yellow fever vaccine supply: A possible solution. Lancet. 2016;387(10028):1599–1600. 10.1016/S0140-6736(16)30195-7 27116054

[21] Lindsey NP, Horton J, Barrett ADT, et al. Yellow fever resurgence: An avoidable crisis? NPJ Vaccines. 2022;7(1):137. 10.1038/s41541-022-00552-3 36323723 PMC9629880

[22] Badio M, Lhomme E, Kieh M, et al. Partnership for Research on Ebola VACcination (PREVAC): Protocol of a randomized, double-blind, placebo-controlled phase 2 clinical trial evaluating three vaccine strategies against Ebola in healthy volunteers in four West African countries. Trials. 2021;22(1):1–15. 10.1186/s13063-021-05572-3 33485369 PMC7823170

[23] Ohimain EI, Silas-Olu D. The 2013–2016 Ebola virus disease outbreak in West Africa. Curr Opin Pharmacol. 2021;60:360–365. 10.1016/j.coph.2021.08.002 34537503

[24] Gouglas D, Christodoulou M, Plotkin SA, Hatchett R. Cepi: Driving progress toward epidemic preparedness and response. Epidemiol Rev. 2019;41(1):28–33. 31673694 10.1093/epirev/mxz012 PMC7108492

[25] Ropero-Álvarez AM, Whittembury A, Kurtis HJ, Dos Santos T, Danovaro-Holliday MC, Ruiz-Matus C. Pandemic influenza vaccination: Lessons learned from Latin America and the Caribbean. Vaccine. 2012 Jan 20;30(5):916–921. 10.1016/j.vaccine.2011.11.092 22155136

[26] Fidler DP. Negotiating equitable access to influenza vaccines: Global health diplomacy and the controversies surrounding avian influenza H5N1 and pandemic influenza H1N1. PLoS Med. 2010;7(5):e1000247. 10.1371/journal.pmed.1000247 20454566 PMC2864298

[27] Ojiako CP. Innovative health financing mechanisms: The case of Africa’s unified approach to vaccine acquisition. Health Policy Plan. 2024;39(1):84. 10.1093/heapol/czad109 37971713 PMC10775217

[28] Nhamo G, Chikodzi D, Kunene HP, Mashula N. COVID-19 vaccines and treatments nationalism: Challenges for low-income countries and the attainment of the SDGs. Glob Public Health. 2021;16(3):319–339. 10.1080/17441692.2020.1860249 33317389

[29] Kunyenje CA, Chirwa GC, Mboma SM, et al. COVID-19 vaccine inequity in African low-income countries. Front Public Health. 2023;11:1087662. 10.3389/fpubh.2023.1087662 36950103 PMC10025287

[30] Kaddar M, Saxenian H, Senouci K, Mohsni E, Sadr-Azodi N. Vaccine procurement in the Middle East and North Africa region: Challenges and ways of improving program efficiency and fiscal space. Vaccine. 2019;37(27):3520–3528. 10.1016/j.vaccine.2019.04.029 31130259

[31] Apeagyei AE, Lidral-Porter B, Patel N, et al. Financing health in sub-Saharan Africa 1990–2050: Donor dependence and expected domestic health spending. PLoS Glob Public Health. 2024;4(8):e0003433. 10.1371/journal.pgph.0003433 39196881 PMC11355530

[32] Nonvignon J, Soucat A, Ofori-Adu P, Adeyi O. Making development assistance work for Africa: From aid-dependent disease control to the new public health order. Health Policy Plan. 2024;39(Suppl 1):i79. 10.1093/heapol/czad092 38253444 PMC10803194

[33] Thomson M, Kentikelenis A, Stubbs T. Structural adjustment programmes adversely affect vulnerable populations: A systematic-narrative review of their effect on child and maternal health. Public Health Rev. 2017;38(1):1–18. 10.1186/s40985-017-0059-2 29450085 PMC5810102

[34] Muckstadt JA, Klein MG, Jackson PL, Gougelet RM, Hupert N. Efficient and effective large-scale vaccine distribution. Int J Prod Econ. 2023;262:108921. 10.1016/j.ijpe.2023.108921

[35] Nomhwange T, Jean Baptiste AE, Ezebilo O, et al. The resurgence of yellow fever outbreaks in Nigeria: A 2-year review 2017–2019. BMC Infect Dis. 2021;21(1): 1054. 10.1186/s12879-021-06727-y 34635069 PMC8504075

[36] Woolsey C, Geisbert TW. Current state of Ebola virus vaccines: A snapshot. PLoS Pathog. 2021;17(12):e1010078. 10.1371/journal.ppat.1010078 34882741 PMC8659338

[37] Emanuel EJ, Buchanan A, Chan SY, et al. What are the obligations of pharmaceutical companies in a global health emergency? Lancet (London, England). 2021;398(10304): 1015. 10.1016/S0140-6736(21)01378-7 34364412 PMC8342311

[38] Noor MN, Rahman-Shepherd A, Siddiqui AR, et al. Socioecological factors linked with pharmaceutical incentive-driven prescribing in Pakistan. BMJ Glob Health. 2021;6(Suppl 3):10853. 10.1136/bmjgh-2022-010853 PMC10175940 36731921

[39] Paremoer L, Pollock A. ‘A passion to change the landscape and drive a renaissance’: The mRNA Hub at Afrigen as decolonial aspiration. Front Public Health. 2022;10:1065993. 10.3389/fpubh.2022.1065993 36518568 PMC9742483

[40] Gavi. African Vaccine Manufacturing Accelerator (AVMA) [homepage on the Internet]. [cited 2024 Jun 25]. Available from: https://www.gavi.org/programmes-impact/types-support/regional-manufacturing-strategy/avma

[41] European Commission. EU support for COVID-19 vaccine manufacturing in Senegal [homepage on the Internet]. European Commission; 2021 [cited 2024 Jan 28]. Available from: https://ec.europa.eu/commission/presscorner/detail/en/ip_21_3562

[42] Seymour S. South Africa: EIB to support increased vaccine production by Biovac [homepage on the Internet]. European Investment Bank; 2022 [cited 2024 Jan 28]. Available from: https://www.eib.org/en/press/all/2022-263-eib-to-support-increased-vaccine-production-in-south-africa-by-biovac

[43] African drugmaker Aspen in talks to manufacture mpox vaccines [homepage on the Internet]. Reuters; [cited 2024 Oct 06]. Available from: https://www.reuters.com/business/healthcare-pharmaceuticals/african-drugmaker-aspen-advanced-talks-manufacture-mpox-vaccines-2024-09-03/

[44] Health Policy Watch. Despite hosting MRNA hub, south africa buys vaccines from India – Highlighting tension between price pressures and local production [homepage on the Internet]. Health Policy Watch; 2023 [cited 2024 Nov 16]. Available from: https://healthpolicy-watch.news/despite-hosting-mrna-hub-south-africa-buys-vaccines-from-india-highlighting-tension-between-price-and-local-production/

[45] Saied AA, Metwally AA, Dhawan M, Choudhary OP, Aiash H. Strengthening vaccines and medicines manufacturing capabilities in Africa: Challenges and perspectives. EMBO Mol Med. 2022;14(8):e16287. 10.15252/emmm.202216287 35758210 PMC9358391

[46] Egypt opens hepatitis B and pentavalent vaccine production line – Health – Egypt – Ahram Online [homepage on the Internet]. [cited 2024 Oct 6]. Available from: https://english.ahram.org.eg/NewsContent/1/1236/492405/Egypt/Health/Egypt-opens-hepatitis-B-and-pentavalent-vaccine-pr.aspx

[47] Abubakar I, Dalglish SL, Angell B, et al. The Lancet Nigeria Commission: Investing in health and the future of the nation. Lancet. 2022;399(10330):1155–1200. 10.1016/S0140-6736(21)02488-0 35303470 PMC8943278

[48] Owolade AJJ, Sokunbi TO, Aremu FO, et al. Strengthening Africa’s capacity for vaccine research: Needs and challenges. Health Promot Perspect. 2022;12(3):282–285. 10.34172/hpp.2022.36 36686053 PMC9808913

[49] Akegbe H, Onyeaka H, Michael Mazi I, et al. The need for Africa to develop capacity for vaccinology as a means of curbing antimicrobial resistance. Vaccine X. 2023;14:100320. 10.1016/j.jvacx.2023.100320 37293248 PMC10244683

[50] Eigbike M. Health research capacity strengthening in low- and middle-income countries: Current situation and opportunities to leverage data for better coordination and greater impact [homepage on the Internet]. 2020 [cited 2025 Mar 18]. Available from: https://tdr.who.int/docs/librariesprovider10/essence/essence-mechanism-consultant-report-2020.pdf

[51] Nigeria Institute for Pharmaceutical Research and Development. National plan for vaccine research and development and local production. Abuja: Nigeria Institute for Pharmaceutical Research and Development; 2023. Unpublished report.

[52] Punch Newspaper. FG to crash drug prices, gets $1bn from AfreximBank [homepage on the Internet]. 2024 [cited 2024 Mar 23]. Available from: https://punchng.com/fg-to-crash-drug-prices-gets-1bn-from-afreximbank/

[53] European Commission. EU and Nigeria boost cooperation on research and learning mobility [homepage on the Internet]. EEAS; 2024 [cited 2024 Aug 04]. Available from: https://www.eeas.europa.eu/delegations/nigeria/eu-and-nigeria-boost-cooperation-research-and-learning-mobility_en?s=114

[54] Wellcome Trust, Biovac, Boston Consulting Group(BCG). Scaling Up African vaccine manufacturing Capacity Perspectives from the African vaccine-manufacturing industry on the challenges and the need for support. 2023.

[55] Kumraj G, Pathak S, Shah S, et al. Capacity building for vaccine manufacturing across developing countries: The way forward. Hum Vaccin Immunother. 2022;18(1):1–17. 10.1080/21645515.2021.2020529 PMC8986212 35086416

[56] WHO Team, Local Production and Assistance (LPA), World Health Organization. A case study on the ecosystem for local production of pharmaceuticals, vaccines and biologicals: The Nigeria context [homepage on the Internet]. Geneva: World Health Organization; 2024 [cited 2025 Feb 16]. Available from: https://www.who.int/publications/i/item/9789240092761

[57] Tekki IS, Nwosu C, Okewole PA. Challenges and prospects of anti-rabies vaccines production in Nigeria. J Vaccines Vaccin [homepage on the Internet]. 2013 [cited 2025 Feb 11];4(8):212. Available from: https://www.walshmedicalmedia.com/open-access/challenges-and-prospects-of-anti-rabies-vaccines-production-in-nigeria-2157-7560.1000212.pdf

[58] World Economic Forum. Regionalized vaccine manufacturing collaborative: a framework for enhancing vaccine access through regionalized manufacturing ecosystems [homepage on the Internet]. 2024 [cited 2025 Feb 19]. Available from: https://www3.weforum.org/docs/WEF_Regionalized_Vaccine_Manufacturing_Collaborative_2024.pdf

